# In Search of Inner Wisdom: Guided Mindfulness Meditation in the Context of Suicide

**DOI:** 10.1100/tsw.2004.17

**Published:** 2004-03-18

**Authors:** Liora Birnbaum, Aiton Birnbaum

**Affiliations:** ^1^ Department of Social Work, College of Judea and Samaria, Ariel, Israel; ^2^ Private Practice in Clinical Psychology, Kfar Yonah, Israel

**Keywords:** inner guidance, meditation, relaxation, guided imagery, suicide, group intervention, bereavement, spirituality, mindfulness, Alaska, Israel

## Abstract

Spiritual concerns are highly relevant, but often ignored, in psychotherapy in general and in suicide in particular. This article presents Internet data and clinical case material bearing on the topic, and describes an innovative therapeutic intervention administered in a group-workshop format with suicide survivors and mental health professionals. The technique incorporates relaxation and mindfulness meditation, with the addition of guided meditation in search of inner wisdom. Results of the group intervention are described and illustrated. Many participants reported a significant positive experience including connection to knowledge that was highly relevant to them in their current state of life. Whether such insights were experienced as coming from within (a deeper part of the self) or from an external source (a guiding figure or presence), indications are that guided meditation can be a powerful resource for therapists and their clients, suicidal and otherwise. Possible applications in diverse populations and settings, as well as the need for further research, are discussed.

## INTRODUCTION

Therapeutic approaches that attempt to incorporate peoples' spiritual beliefs and practices into clinical care increasingly have become a focus for investigation in recent decades. One such widely researched technology in the field of human development is meditation. Positive effects of meditation on physiological (e.g., heart disease, hypertension) and psychological problems (e.g., anxiety, depression, addictions) are well documented[1,2,3]. Spiritual therapeutic approaches are, however, still relatively rare in the prevention, rehabilitation, and research efforts in the specific area of suicide.

Traditional approaches to the phenomenon of suicide have attempted to identify its various causes and types, in order to advance assessment, treatment, and prevention efforts. Risk factors that have been identified in suicide include previous suicide attempts; history of suicide in the family; personal history of mental disorder, violence, abuse, or incarceration; and current depression, substance abuse, life stress or loss, social isolation, serious medical or psychological condition, or availability of lethal means; and recent suicide among social models or peers[4,5]. Mood disorders, particularly depression, are clearly linked to suicide. Beck et al.[6] have identified three cognitive factors predictive of suicide risk in depression: negative view of the self, the world, and the future. Suicidal individuals often exhibit “tunnel vision” of a particularly pessimistic kind, seeing no options for positive change. The abyss of the depression seems enormous and unending, and suicide may seem to be the only way out. Often this condition is accompanied by low self-esteem and low self-efficacy, feelings of worthlessness or guilt, and it may be engendered by a variety of loss experiences: loss of health, job, or function; loss of love, relationship, or significant other. Assisted or unassisted suicide in the context of terminal illness is usually distinguished from other types. Further distinctions are often made between suicidal behavior in neurotic individuals, a “cry for help”, repeated suicidal acting out in borderline personalities, and suicide resulting from delusional beliefs in psychotic individuals. Some differentiate between suicide emanating from a desire to kill the body and suicide resulting from a desire to escape from an impossible situation[4,5].

Existing approaches to assessment of suicide have focused on identifying risk factors as described above, and applying treatment accordingly: high-risk individuals may be hospitalized and/or medicated, and efforts made to increase social support (starting with constant monitoring until the suicidal state passes). Crisis intervention efforts often aim for possible situational change, while longer-term therapeutic efforts usually target the depression or problems dealing with relevant losses, stress, guilt, etc., whether from an individual or a systemic perspective. Despite laudable prevention efforts, suicide remains a leading cause of death and a devastating event to the victim’s social system[4,5,6].

We posit that peoples’ choice to live or to die is highly related to aspects of their spiritual life. This holistic view suggests that in order to help a person who has chosen to die, we need to evaluate all aspects of their life, including mystical and nonrational aspects. Our current scientific understanding of suicide may lack highly relevant information in the lives of suicidal individuals, simply because it is not amenable to traditional, rational approaches to research and academic discourse.

In fact, we might go so far as to say that suicide may be considered a spiritual illness, requiring spiritually relevant practices as an integral part of assessment and treatment[7]. Such metaphysical dimensions might help us grapple with a behavior that is so fundamentally opposed to basic physical laws of self-preservation. Aboriginal elders and Medicine People, for example, have attributed the alienation from self in depression to the overwhelming materialism of the modern world, which prevents us from seeing ourselves as we really are and obstructs our natural groundedness and spirituality[8].

The dearth of writing on the topic of spirituality and suicide may be related to the view that prevailed until the 1990s in the mental health fields, that spirituality is necessarily and directly linked to religion[9]. More modern approaches to the subject emphasize that while religion and spirituality may be related in certain people, the two are clearly far from synonymous. Canad’s definition of religion refers to “an integrated belief system that provides principles of behavior, purposes of existence, meaning of death, and an expression of reverence for a supernatural being (or beings)”[10, p. 37]. Spirituality encompasses an individual’s perception of the sacred, their ultimate values, beliefs, and relationship to both the natural and metaphysical worlds[9,10,11,12,13,14,15]. Jacobs[16, p. 172] defines spirituality as “heart knowledge where wholeness, meaning and inner peace occur [along with] a sense of being at one with the inner and outer worlds.” The significant difference between the two is that religion is a structured form of spirituality that usually has a group following. Spirituality may or may not involve a structured belief system or group following, while it expresses human longing for a sense of meaning, fulfillment, and connection with something bigger that transcends ordinary life experience[7].

Sometimes, different forms of spirituality may determine life or death. Various native populations, including the Alaskan Inuit, the Maori of New Zealand, Native Americans, and Native Australians, all exhibit high suicide rates[19]. However, Native Americans connected and committed to native (non-Christian) cultural spirituality exhibit significantly less suicidal behavior than their peers[20]. Native identity crisis and the cost of being caught between two cultures can help to explain this phenomenon[21].

The first section of this article attempts to clarify and provide partial and tentative answers to some central questions bearing on spirituality and suicide: What spiritual world exists behind suicidal ideation?

What is the nature of the spiritual values, beliefs, and concerns held by suicidal individuals, and can these be channeled in healing directions? What do we know about the spiritual issues of people who are physically in life, but psychologically are caught between life and death?

One potent source of information about clinical and nonclinical samples of people contemplating suicide is the Internet. There are currently dozens of active sites relevant to suicide in cyberspace. This phenomenon has become a source of some concern of late, since a preliminary National Institute of Mental Health (NIMH)[22] study of suicide sites found several suicide prevention sites, but many more "how-to" sites. Suicidal individuals converse through a variety of forums and chat groups on these sites. We obtained data from an Internet forum in which people who defined themselves as suicidal corresponded about their experiences[23]. One participant raised the question: "What do you believe that a suicide attempt means on a spiritual plane?" Thirty responses containing spiritually relevant statements were collected and content analyzed. Clinical material illuminating the life stories of several individuals in the process of becoming free of suicidal ideation supplements the Internet data in our attempt to gain an initial foothold in the territory of spirituality and suicide.

The second part of the article presents findings from participants in an experiential workshop on guided meditation and connecting to internal wisdom, presented by the first author at a conference on suicide recently held in Alaska[24]. Most mental health clinicians in Alaska today are White, while most suicide attempters are Native, and spiritual differences between the two reinforce the image of White man fixing the Native[25]. The workshop data serve to further clarify issues around spirituality and suicide, and illuminate powerful potential resources for suicidal (and other) individuals.

We do not attempt to prove or disprove any theoretical assumptions here. Our goal is simply to share this type of work with other professionals, in order to encourage and sharpen the focus on the significance of the spiritual dimension in empowering clinicians and clients in the healing process.

## SPIRITUAL PERCEPTIONS AND BELIEFS

"What do you believe that a suicide attempt means on a spiritual plane?" The fact that this question was raised spontaneously in a general suicide site and the numerous reactions it engendered shows that people in a suicidal state are highly involved with spiritual concerns. Several common themes emerged in the responses:

1. **Evaluations of the suicidal self:** Coholic[17] notes that a connection to spirituality often converges with experiencing a positive sense of self. In contrast, a profound lack of self-love and self-worth often accompanies suicidal ideation, as reflected in these responses: "It is a way of saying that you regret being created." "Perhaps I should give someone else a try."
2. **Belief in a higher power:** This theme often appeared along with the idea of judgment of actions by God. Indeed, suicide is seen as a sin deserving of punishment in a variety of religions. "It is an unpardonable act against a higher power." "I felt that God would not forgive me because I was making the assumption that I could create someone better than he could."
3. **Belief in an on-going cycle of life and death:** The intention to preserve the essence of oneself for a better life after death was identified by Maltsberger and Bui[26] as a fantasy. However, some people view the concept of reincarnation as a real possibility and a natural part of the life cycle. "What suicide means is I know I'll be back and I'd like a fresh new start." "Now I see hell as mental state. We bring our guilt, anger, sadness and depression to the afterlife and that creates our hell."
4. **Perceived relationship with God:** Numerous examples illustrated how peoples' relationships with God may parallel intimate relationships with significant or parental figures, often involving feelings of love and trust: "The two people who know me best are God and my therapist." "I feel God is the only person I can trust on this issue." Other examples focused on anger: "I felt that God had a lot to answer for, and in some petty way this seemed to justify the defiance I felt." Still

> others reflected a perception of guidance: “I survived. That is God’s way of telling me this is not the time. He will reveal his guidance to me in a manner I can handle.”

In some cases, the relationship seemed highly ambivalent, combining longing for comfort and love with blaming God. Such ambivalence might be especially common in those adolescents who are prone to building up misunderstanding and resentment toward their parents from early childhood[27]. Indeed, according to NIMH[22], Internet suicide-site-users are often young people who are troubled by bullying peers, romantic breakups, or abusive family members.

Michel, for instance, is an adolescent who tried to kill herself twice. She expressed much anger about her life: “I felt God wanted to punish me for something I had done in a previous life. He gave me a manic-depressive mother who kidnapped me, and a drunk father, who never realized his friend was molesting me. Over the years, I never met a therapist I did not hate. All they were interested in was to fill me up with drugs. The only thing that pulled me from the path I laid out for myself was to change my own attitude. I convinced myself that this life — I chose to live, so I could gain insight into the heart and minds of people less fortunate and those who are morally corrupted. This outlook has brought me to a place where I feel empathy for others, without pain.”

This example demonstrates that a negative cycle that dominates one’s life (lives?) can be broken. Michel broke it by choosing to accept what life had brought her and to find significance in her remaining alive. By doing this, she moved from a state of helplessness to a state of perceived control and commitment. This, in turn, allowed her to let go of her anger and the victim role, and to take responsibility for her life.

Michel’s story, among others, helps illustrate that spiritual beliefs represent a powerful source of resilience or despair; they drive self-interpreted concepts of meaning, purpose, and responsibility for one’s choices in life. Hartman[28] describes finding meaning in crisis situations as spiritual work involving spiritual goals. Coholic[17] found that most of the social workers she studied defined spiritually informed treatment as involving helping the client find meaning. However, she disputes the assumption that one can always simply find meaning in suffering, stressing that sometimes it has to be constructed. Some clients have less readiness to engage in meaning-making and some take longer to achieve meaning than others. In fact, some never succeed. But in general, people can make sense of their suffering and such meanings can help them cope.

History teaches that people who fail to find meaning in traumatic events often abandon their spiritual beliefs. Many Holocaust survivors, for example, lost their faith in a guiding higher power because they could not contain the extreme dissonance between such beliefs and their tremendous exposure to pain, suffering, and death. It seemed that “if God existed, He never could have allowed such things to occur.” The idea that there might be a purpose behind all that suffering cannot be perceived rationally, but a spiritual approach posits that all events have their purpose[29]. Indeed, traumatic circumstances like the Holocaust have also led to opposite, belief-strengthening effects; in the absence of home, food, clothing, and family, sometimes all that was left was to cling to one’s spiritual beliefs. Painful circumstances can motivate spiritual processes that lead a person to observe their life and their real needs more honestly. Laufer[30] found that the more Israeli youths were exposed to terrorist attacks, the more they showed not only adverse symptoms of trauma, but also positive psychological growth. Tedeschi and Calhoun[31] provide a measure of such growth, the “Post Traumatic Growth Inventory.” Two of the five dimensions incorporated in the inventory are clearly spiritually relevant (“spiritual change” and “appreciation of life”). Laufer[30] saw traumatic events as shaking the individual’s cognitive assumptions, causing a rethinking that in some cases leads to a positive shift in the view of self, others, and the world. Spiritual approaches assume that life is not a series of random occurrences, but of meaningful events and choices. Accepting this can allow moving from an existential position of judgment of life (and of self) to a position of observing it, in order to understand the underlying purpose of events. As Holocaust survivor and advocator of man’s search for meaning, Victor Frankl[32], put it, “It doesn’t matter what we expected out of life, only what life expected of us.”

## THE OBSERVING SELF

The ability to observe our "selves" and our lives is acquired, and entails listening and tuning in to ourselves and the world around us. Eastern spiritual traditions incorporate holistic conceptions of healing and intuitive mind-body-soul interconnections. The role of the body is to host the soul and provide it with a home (Buddhism and Judaism), while the role of the soul is to learn and develop through experienced life events and decisions. From this point of view, suicidal thinking may be seen to involve negative connections between mind and body, and disconnection from the spirit. Negative thinking in the mind such as suicidal ideation certainly has the power to negatively influence perceptions and feelings relating to the body. The mind and the soul may be said to each have their own voice. The observing self is better able to listen and identify the different voices that exist within and around it.

The external voice: We identify the external voice as that of the mind or ego. The ego contains our ambitions, needs, and goals, which are the result of evaluations and value attributions emanating from our environment, i.e., significant others. This external voice is important. It accompanies us throughout life and assists us in defining who we are, understanding ourselves, and how we function in the world. It develops right along with us and is therefore inextricably connected to our subjective experience and our "self-concept". As such, it may take on a variety of roles representing various aspects of our personality: the aggressor, the knower, the innocent, the guilty, or the rebel. Indispensable as it is, this is also the voice behind suicidal behavior. The following examples are taken from private practice with suicidal clients.

Nellie (14) swallowed bleach in response to the "voice" that told her "to evaporate and disappear" after her mother remarried — in order that she not get in the way. (Her biological father had physically abused the family before the divorce.) When her anorexia led to cessation of her menstrual periods, it turned out that Sharon (18) was following a "voice" that said "the less of you there is — the less place you'll take up in the family, and the less pain you'll feel." (Her family had gone through a difficult divorce, including emotional abuse.) The severe depression experienced by Susan (23) also related to a "voice" that told her "anyway, there's no point in living since nobody sees or hears you." Such voices are not delusional, but can be confusing and upsetting because they are perceived as providing desperately needed guidance and solutions to impossible life situations. They are also experienced as coming from within, since we know them so well and for so long — and thus they may easily obscure our own authentic inner voice.

The inner voice: We treat this voice as that of inner guidance, inner wisdom, or intuition, which may serve as a direct and immediate source of knowledge, independent of conscious ego-function and sense perception[33]. Linehan[34] has described the presence of "wise mind" even in severely disturbed clients. This part of the person, or this state that a person can access, is precisely what provides the necessary potential for positive change. As we use the term inner wisdom, we speak of a deeper level of consciousness, in which the person is directly in touch with authentic needs, desires, and values at the core of their true self. This level of wisdom lies in quietude, far beneath the changing and sometimes raging seas at the surface of our conscious existence. Through connection to this inner wisdom, one may know "deep down" what is right for them, what they need, and what is their higher good, both in general as well as in any particular situation. This connotes the person's ability to recognize a sense of their mission or their direction toward self-actualization, which in turn refers to realizing more of one's inner potential expressed in any and all areas of life[35]. One of the main obstacles to progress on this road is accepting the prices to be paid for approaching this ambitious goal.

Dorothy (45), married mother of two, is a successful psychotherapist and tenured professor with numerous publications. Despite her success, Dorothy has long suffered from extended episodes of severe depression, requiring antidepressant medication. In addition, she has twice been diagnosed with cancer and recently also with a chronic bowel condition. She was unable to make sense of her depressive episodes and her escalating health problems only depressed her further. More than once she expressed that she "felt closer to death than to life." Guided meditation assisted her in getting in touch with her inner wisdom. Only then was she able to identify her drive to publish as an addictive process, symbolically fulfilling her need for additional pregnancy and birth experiences.

Dorothy's case also illustrates how somatic symptoms may be related to unspoken psychospiritual dilemmas. When balance is lacking, each voice dictates a different path, and the choice between them may correspond quite literally to a choice between life and death. Conflict between internal and external voices is an existential necessity. One goal of spiritually sensitive therapy is to clarify and foster a dialogue, while facilitating a search for balance between the different voices and the needs they represent[36].

## MEDITATION AS A THERAPEUTIC INTERVENTION

In order to facilitate learning about the inner voice, an experiential workshop was developed. Its goals included experiencing inner quiet, expansion of consciousness, and listening to the voice of inner wisdom. The methods selected were mindfulness meditation followed by guided meditation.

The meditation literature describes many different meditative practices. Reviews of this literature identify two basic types of meditation practice: concentrative meditation and mindfulness meditation (also known as "opening up" or insight meditation). Concentration practices focus on a specific object of attention such as awareness of the breath, a candle flame, or a repeated mantra. During the technique, the individual's awareness settles down and experiences transcendental consciousness where it is open to itself[37,38].

Perhaps the most significant clinical application of mindfulness meditation is the capacity to adopt an "observing self". The person is instructed to be aware of any mental content, including thoughts, imagery, physical sensations, or feelings as they occur in consciousness on a moment-to-moment basis. Mindful awareness is based on an attitude of acceptance. Rather than judging as good or bad, healthy or sick, worthy or unworthy, mindfulness accepts all personal experiences as simply "what is" in the present moment[39]. Therefore, before beginning the mindfulness exercise, participants were urged not to judge, but to simply observe their experience in order to learn about themselves.

This observing self is also what connects meditation, as part of psychotherapy, to behavioral or cognitive techniques such as self-monitoring or identifying dysfunctional thoughts. But there is a big difference. In meditation, we coach people to treat their passing thoughts as "just thinking" instead of as personal directives coming from the self. Meditation can help us to dis-identify from our thoughts (including suicidal ideation) and can loosen their compulsive hold over us[39]. Teasdale et al.[40] studied mindfulness meditation as a relapse prevention treatment for depression, and illustrated how it differs from cognitive therapy when applied to negative thoughts like "My life is a failure; I am miserable and see no reason to go on living." A cognitive therapist might try to help the client dispute the content of such thoughts, perhaps by suggesting counterexamples to minimize overgeneralization. In contrast, the aim of meditation therapy is not to change the thought itself, but to alter the client's attitude or relationship to it. Thinking that my life is a failure is accepted as just a thought that occurred in the mind. Our participants were asked to pay careful attention to the passage of thoughts through their minds so that they could detect when and how a different voice started voicing itself. They were asked to focus on the turning point, where familiar thoughts and sensations turned different or new.

## THE WORKSHOP METHOD

### Participants

Workshop attendees were self-selected from the 300 mental health professionals and six lay persons attending a conference on cross-cultural perspectives in suicide held at Nome, Alaska[24]. Enrollment in each of the two workshop sessions was limited to 20 people, and some people needed to be turned away. All 40 participants chose this experiential activity during the specified time slot instead of attending another of several theoretical presentations that day. Ages of the 25 women and 15 men ranged from 25–60 years. Of the six nonprofessionals at the conference, 5 (4 Native, 1 White) chose to attend the workshops. These were lay members of Alaskan rural communities who had a history of one or more suicide attempts, but none were actively suicidal at the time of the workshop. There were 35 (15 Native and 20 White) mental health professionals from northwest Alaska, with experience working with suicide in the community: village-based counselors (12 Natives), school counselors (3 Natives, 7 White), family therapists (5 White), social workers (4 White), psychologists (3 White), and (1 White) psychiatrist.

## Procedure

The workshop included several stages: general relaxation, mindfulness meditation, and a guided meditation seeking connection with inner guidance. The relaxation involved breathing, finding an inner center, and releasing tension in the various muscle groups. The mindfulness meditation asked participants to notice any and all thoughts, feelings, and body sensations (in and around the body), and simply to observe them. While in deep relaxation, connection to inner guidance was facilitated via visualization of a safe place, followed by instructions to invite or open up to inner guidance (or wisdom). Just as the safe place could evolve according to personal preference, participants were reminded that guidance or wisdom could appear in various forms, including an image, presence, figure, message, or intuitive knowledge. If they noticed a figure or presence, they were asked to connect to them and receive any relevant message, advice, or image which seems right at this point in their life. If no presence was noticed, they were encouraged to imagine how one might appear and what message it might have brought. Based on results of a pilot workshop, we decided not to have participants fill out a structured questionnaire — in order to avoid imposing rational organization on the experiential data. Rather, participants were simply asked to describe their experience or reactions (images, thoughts, feelings, or body sensations) in writing. These responses, along with transcribed participant comments in the concluding group processing, were subjected to qualitative content analysis, which employed inductive ground theory methods to extract significant themes[41].

## RESULTS

Most participants (27) reported meeting or feeling some sort of presence. For 6 of them, this was not the first such encounter. The experiences were distributed approximately evenly between two basic types: tangible vs. abstract. Illustrations of the tangible variety include: “My [late] mother, father and brother…came to me with open arms.” and “A bright image of me was above me.” A typical response of the latter sort: “There was some kind of undefined presence near me, which emanated a clear energy of love, acceptance and compassion for me.” The dichotomy presented above did not hold universally; some participants felt a distinct presence of a human figure, yet could not identify or describe the person. Ten participants described a change in the temperature or energy they felt around or inside their bodies.

Among those who experienced meeting an “empowering” and “loving” figure, 5 described difficulty or unwillingness to separate from them on termination of the meditation. They described the separation as painful and said they wanted more time. Twenty reported receiving the message from the figure that they were always available to them, e.g.: “I know now that I’ll always be able to go back and meet him when I need to.”

Interaction with the guiding image (or wisdom) was variable. Some entailed verbal interaction with a distinct message: “The time has come for you to separate from your anger and find a partner in life.” Others experienced dialogue in questions and answers: “Q: What is your role? A: I am your spiritual guide, and I will guide you when your time comes to leave the world.” Some reported meaningful visual images: “I found myself in a pool of water, with many other people, of all races and cultures, smiling at each other. I got the message that I am equal to all, and not less than anyone in this world.” Others described an inner knowing that was telepathic, such as “I am very beloved.” Of the 13 who did not experience a presence, 4 attributed this to high control needs, inability to relax, or difficulty with internal imagery. Several reported that they felt benefit only from the relaxation and meditation. One participant declared she would never try meditation again, after visualizing black, scary eyes looking angrily at her.

Fascinating comments on the process were collected and will be described elsewhere (Birnbaum, in process); the following three examples are taken from the experiences of those who had made at least one suicide attempt. It is worth noting that all these individuals had previously found their way to meditative work, whether alone or in therapy, and were already familiar with their inner guidance.

Gina, a 42-year-old Native Inuit and artistic mother of two, experienced severe emotional and physical abuse in her adolescence and later in her marriage to a violent husband. Fairly soon after her suicide attempt, she made contact with her inner guidance, which she experienced as a male figure who encouraged her to trust him. In the workshop, she received the message from him that she needed to change things in her life and to engage in writing and drawing. This information was accompanied by images of a boat arriving to a harbor and unloading a heavy cargo of paper, which was meant for Gina's artwork and the telling of her life story.

John, a 45-year-old Native and father of three, was the victim of sexual abuse at the hands of his classmates over the course of years. He never told anyone about the abuse. Soon after marrying, he became dependent on alcohol and his family of origin cut him off. When his first son was born, he became depressed and suicidal. John entered long-term psychotherapy in which he learned meditation techniques. His safe place was on a beach, beside a burning fire. He experienced his guiding figure as a polar bear whose spirit had accompanied him from birth. His guide taught him to observe the fire, wood, and ocean and to learn about his feelings, mood, and current difficulties from the way they behaved. The actual messages sprang from the distinct beach imagery in every meditation. In the workshop, the calm beach scene was replaced by an image of a raging sea, and the burning fire alternately extinguished and reignited. At first John was puzzled, but his guide provided the message that John was worried about something and needed to continue meditating in order to clarify the issue. For John, this avenue provides a singular sensation of someone being connected to him and recognizing his emotional state, which he experiences as an important source of inner support. The stance of mindfulness he has attained via meditation allows him to remain in a calm state of objective detachment, simply observing the turbulent and disturbing images arising from within, without undue distress.

Gary, a 30-year-old White man, was especially touched by his meeting with an image he knew from previous meditations: "… a lady in her 50's wearing white, with long hair and glasses… The feeling of occurrence evenly distributed itself through my entire body. I felt calm. The agitation, worry, distress vanished and, not like the usual disappearance of feelings, I felt or rather knew that everything would be and already was all right." She told him that "he is at the right place, doing the right thing" coming to therapy. He went on to say that "Since [meeting her] — I have never felt alone and I know I am not. It's like I have arms wrapped around me."

Personal interpretations: Who is the figure that appears in meditation and provides messages or advice? All 21 people who experienced this type of meeting for the first time were mental health professionals. As described above, some identified the figure as a lost relative (parents, siblings). Others saw the figure as an unfamiliar entity whose express purpose was to guide them. Of the 6 who had experienced this type of meeting before (3 professionals, 3 suicide attempters), some felt the figure to be external, while others experienced it as a manifestation of their higher self.

Serdahely[42] discusses whether the presence people report in near death experiences is their higher self or an external (female) figure whose purpose is to provide consolation, especially to those abused as children. Such reports provide interesting ideas about the possible structure of spiritual worlds, with various roles designed to assist in specific situations. We have discovered that people who experience the same presence in their meditation over time develop a variety of thoughts and feelings about and toward the figure. Usually these revolve around love and connectedness, but further research is needed to enhance our understanding of this presence and peoples' inner relationships with it.

## DISCUSSION

We have presented data and described an intervention which seems to have potential for helping people in the context of suicide. Diverse traditional psychotherapeutic approaches would deal differently with a suicidal or post-suicidal individual. Behavioral approaches might focus on manipulating environmental contingencies and both primary and secondary reinforcers for related behavior. Cognitive approaches might focus on dysfunctional thought patterns (underlying assumptions, automatic thoughts, schemas) and attempt to dispute those supporting suicidal thinking. Psychoanalytically informed treatment might focus on interactions with significant others in early childhood that have bearing on the patient's suicidality. Integrative therapies might combine various elements of these and other approaches with particular clients and their specific problems. Systemic approaches would emphasize the need for looking at the entire system in the assessment and treatment of the suicidal family.

The results of the workshop described here demonstrate that such an experiential activity may be utilized as a cross-cultural spiritual intervention for post-suicidal individuals, as well as for clinicians. The fact that professionals and lay people signed up so readily reinforces our impression from the Internet data that spiritual issues are potent and salient in the context of suicide. The workshop effectively bridged a variety of differences between participants, including those between Whites and Natives, clinicians and clients, socioeconomic status, age, and gender. A majority of participants experienced strong feelings of love, compassion, acceptance, and a sense that "we are all connected." The fact that such feelings emerged in a single session with a group of strangers indicates powerful psychospiritual therapeutic potential. Therapists who have had such an experience themselves are likely to be more open and less skeptical about clients' spiritual reports. Such a workshop may especially empower post-suicidal participants, who effectively become teachers as they show clinicians and other clients how practiced spirituality helped them re-choose life. Obviously, suicidal individuals may be more open to learning from similar others, who become effective models for constructive behavior and hope.

Such an experiential intervention may be appropriate for actively suicidal individuals and people at risk of becoming suicidal; for families of suicide victims, suicide attempters, and suicidal individuals; and for the bereaved in general. While the group application demonstrated here is cost effective, mindfulness meditation may be applied to individuals, couples, or families, as well. It can be used on its own as the treatment of choice, as one component in a treatment package, or as an adjunct to virtually any treatment modality. One may easily extend behavioral relaxation techniques to include mindfulness. Cognitive therapy may be transformed to metacognitive therapy by adding or shifting the focus to thoughts about the thoughts. Deeply buried memories and associations may become available through the process of meditation for analysis in psychodynamic psychotherapy. Thus, on-going mindfulness meditation can enable significant processes of self-transformation. As we become attuned to our internal wisdom, we can discover hidden aspects of our self. Different wishes and goals, rearrangement of priorities, a sense of connectedness with others, and positive changes in interpersonal relationships — all become likely. The practice of mindfulness meditation may well lead to the emergence of a fundamentally different sense or perception of self.

Obviously, application in various potential combinations of formats, settings, dosages (length, frequency, and total number of sessions), and therapeutic approaches, and possible benefits or problems with different clinical and nonclinical populations need to be studied in future qualitative and quantitative research, along with maintenance of effects over time. The qualitative information presented here, of a single session with a small sample in a specific setting, is insufficient for drawing theoretical or practical conclusions on the topic.

As we have described, clinical experience and the literature[43] show that the client's spiritual world is not present enough in the therapeutic space. When therapists do not speak the same spiritual language as their clients (or any spiritual language), they are at risk of ignoring an important resource for resilience — which, in the case of suicidal clients, just might be what keeps them alive.

Various issues present themselves regarding meditation as a therapeutic intervention, including: In what way is the therapist role influenced by this method? How might it impact the therapeutic relationship? Is there a potential for harm in using it, with whom, and in what situations? We recommend that therapists who choose to use guided meditation with clients be connected to their own inner wisdom. Familiarity with spiritual guidance may come in handy in cases of unexpected or difficult client reactions. For example, a client may report getting an internal message to carry on with his suicidal plan. The therapist's own knowledge and experience would tell him that true inner guidance is exclusively loving and universally geared towards choices and coping strategies that promote life, not death. Professionals working with populations who stand to benefit from such work, but who do not share these beliefs should probably consider a referral to someone who can speak this language.

Regarding client selection, we do not currently recommend involving actively psychotic people in such work, since it is based on one's ability to listen to authentic messages. Pre- or post-psychotic clients, borderline level of personality organization, or current risk of decompensation would all be contraindications. We counsel caution in applying such methods to dissociative personality disorders and recommend that only clinicians experienced in treating such disorders consider doing so. We have as yet no data on the effects of psychotropic or other medication on the process or on maintenance of effects. Based on our experience, a small percentage of people (perhaps, though not necessarily, with high levels of latent disturbance) may experience intimidating images or messages, like those described above in one workshop participant. People with high control needs may feel frustrated at not being able to relax or connect to deeper parts of themselves. In our clinical work, we encourage such individuals to continue meditating, focusing on the ability to "let go of control." More care in assessment, preparation, and clarification of expectations as well as a slower, more flexible approach with the technique may be indicated for all such clients. They may require a more solid therapeutic relationship and a warmer holding environment to risk letting go, as well as good self-soothing skills in case they encounter disturbing material.

As for the therapeutic relationship, a unique alliance may develop when therapist and client meditate together, allowing optimal dialogue based on equality. The clinician can use his or her own guidance in this process to gain better understanding of the client's needs. Additionally, it is not uncommon for therapists to get sucked into the helplessness that suicidal clients tend to project. Connecting to their inner guidance can help the helper hold on to the hope and belief in the power of life until the client is ready to own them himself.

## CONCLUSION

Guided meditation and connecting to inner wisdom may be powerful therapeutic techniques. Further investigation is required to increase our understanding of the nature of guiding images and level of receptiveness to them, demonstrated by people of diverse cultural, religious, and personality characteristics. Exploring patterns or stages in the development of the relationship with a spiritual guide in different phases of the life cycle is yet another area for future studies.

Integrating spirituality into therapy is like helping to build an internal shrine. We have often discovered that the shrine was already there; the door simply needed to be opened.

## BIOSKETCHES

**Liora Birnbaum, Ph.D.,** is a Certified Family Therapist. She is Assistant Chairperson of the Department of Social Work and Head of the BA program in Social Work at the College of Judea and Samaria, Ariel, where she teaches clinical courses. She also has a private psychotherapy practice in Kfar Yonah, Israel. E-mail: labirnbaum@bezeqint.net

**Aiton Birnbaum, Psy.D.**, is a Licensed Clinical Psychologist and EMDR Consultant in private practice in Kfar Yonah, Israel, specializing in the treatment of trauma and bereavement. He is a Lecturer in the Depatment of Behavioral Sciences, College of Judea and Samaria, Ariel. E-mail: labirnbaum@bezeqint.net
